# Movement Imitation Therapy for Preterm Babies (MIT-PB): a Novel Approach to Improve the Neurodevelopmental Outcome of Infants at High-Risk for Cerebral Palsy

**DOI:** 10.1007/s10882-019-09707-y

**Published:** 2019-11-18

**Authors:** Marina Soloveichick, Peter B. Marschik, Ayala Gover, Michal Molad, Irena Kessel, Christa Einspieler

**Affiliations:** 1 Neonatal Intensive Care Unit, Lady Davis Carmel Medical Center, Haifa, Israel; 2 The Ruth and Bruce Rappaport Faculty of Medicine, Technion, Haifa, Israel; 3 Research Unit iDN - interdisciplinary Developmental Neuroscience, Division of Phoniatrics, Medical University of Graz, Graz, Austria; 4 Department of Child and Adolescent Psychiatry and Psychotherapy, University Medical Center Göttingen, Göttingen, Germany; 5 Leibniz ScienceCampus Primate Cognition, Göttingen, Germany; 6 Center of Neurodevelopmental Disorders (KIND), Department of Women’s and Children’s Health, Karolinska Institutet, Stockholm, Sweden

**Keywords:** Cerebral palsy, Early intervention, Intraventricular haemorrhage, General movements

## Abstract

To improve the neurodevelopmental outcome in infants with high grade intraventricular haemorrhage and cramped-synchronised (CS) general movements (GMs). Four very preterm infants with intraventricular haemorrhage grade III (*n* = 3) or intraventricular haemorrhage with apparent periventricular haemorrhagic infarction (*n* = 1) were diagnosed with CS GMs at 33 to 35 weeks postmenstrual age. A few days later MIT-PB [Movement Imitation Therapy for Preterm Babies], an early intervention programme, was commenced: the instant an infant showed CS movements, the therapist intervened by gently guiding the infant’s limbs so as to manoeuvre and smoothen the movements, thereby imitating normal GM sequences as closely as possible (at least for 10 min, 5 times a day, with increasing frequency over a period of 10 to 12 weeks). After a period of consistent CS GMs, the movements improved. At 14 weeks postterm age, the age specific GM pattern, fidgety movements, were normal in three infants, one infant had abnormal fidgety movements. At preschool age, all participants had a normal neurodevelopmental outcome. This report on four cases demonstrates that mimicking normal and variable GM sequences might have a positive cascading effect on neurodevelopment. The results need to be interpreted with caution and replication studies on larger samples are warranted. Nonetheless, this innovative approach may represent a first step into a new intervention strategy.

## Introduction

Infants born very preterm (<32 weeks’ gestational age) are at increased risk for adverse neurodevelopmental outcome as a result of prenatal or perinatal brain lesion (Patel 2016; Valdez Sandoval et al. 2019). Although the rate of infants developing severe cerebral palsy (CP) has decreased during the last years (McGowan and Vohr 2019), very early preterm birth is still a risk factor for this outcome diagnosis (Burnett et al. 2018; Spittle et al. 2018). According to the EPIPAGE-2 cohort study, the overall rate of CP in children born between 27 and 31 weeks’ gestation is 4.3% (Pierrat et al. 2017). Large cohort studies showed that 28 to 30% of very preterm infants with intraventricular haemorrhage (IVH) grade III and 60% of individuals with intraparenchymal haemorrhage (IPH) (Volpe 1998) were later diagnosed with CP (Ancel et al. 2006; Bolisetty et al. 2018; Radic et al. 2015).

The Prechtl general movements assessment (GMA) (Einspieler et al. 2004; Einspieler and Prechtl 2005) enables us to assess the young nervous system reliably, sensitively and non-intrusively during preterm age until about 5 months post-term. Abnormal general movements (GMs) are predictive of an adverse neurological outcome, in particular, of CP (e.g., Prechtl et al. 1997; Einspieler et al. 2013; Bosanquet et al. 2013; Novak et al. 2017). It is mainly the pattern of cramped-synchronised (CS) GMs that predicts spastic CP (with a positive likelihood ratio of 45) if consistently present for several weeks (Ferrari et al. 1990, 2002, 2011; Einspieler and Prechtl 2005; Bruggink et al. 2009; Yang et al. 2012). CS GMs appear rigid and lack the normal smooth and fluent character that characterises normal GMs; limbs and trunk muscles contract almost simultaneously and then relax almost simultaneously (Einspieler et al. 2004; Einspieler and Prechtl 2005). The age of appearance of CS GMs has been associated with the degree of the eventual functional impairment caused by CP: the earlier CS GMs appear, the greater the functional impairment (Ferrari et al. 2002), though CS GMs were hardly observed before 32 weeks (Einspieler et al. 2015).

Based on what we know from a number of studies (e.g., Ferrari et al. 2002; Bruggink et al. 2009; Yang et al. 2012; Morgan et al. 2016a; Kwong et al. 2018), one would expect a significant neurodevelopmental deficit if a preterm infant presents with severe structural (e.g. high grade IVH) and functional impairments (e.g. CS GMs over a period of at least 3 consecutive weeks). Such early identification cries out for early intervention, but we only know little about the effect of intervention that already starts preterm (Hadders-Algra 2014; Silveira et al. 2018; Hutchon et al. 2019).

Considering the high risk of adverse neurological outcome due to severe brain injury accompanied by CS GMs and the inconclusive results of studies on very early intervention, we intended to find out whether a specifically designed intervention, aimed at superimposing fluent and variable GM sequences (i.e. therapist-mediated) has a positive effect on the neurodevelopmental outcome.

## Methods

### Case Histories

From June to December 2012, four very preterm infants (born at 27 to 28 weeks’ gestation), two males and two females, were admitted to the Neonatal Intensive Care Unit at the Carmel Medical Center, Haifa, Israel. Their cranial ultrasound abnormalities revealed high grade IVH: IVH grade III or IVH with apparent periventricular haemorrhagic infarction (APHI) according to Volpe (2018) in all four infants. Repeated GMA demonstrated CS GMs from 33 or 35 weeks onwards. The infants’ clinical data are listed in Table 1; none of the infants received anticonvulsants. All parents gave their informed consent for assessment, intervention, and publication of the results; the ethical committee of the Carmel Medical Center Haifa approved the interventional approach.

**Table 1:** Clinical data of the four cases

	Case 1	Case 2	Case 3	Case 4
Gender	Male	Male	Female	Female
Gestational age at birth in weeks^+days^	28^+4^	28^+4^	27^+2^	28^+0^
Birth weight in grams	1231	1148	1000	1000
Plurality	twin^a^	twin^a^	twin^b^	singleton
Mode of delivery	caesarean section	caesarean section	spontaneous vaginal delivery	spontaneous vaginal delivery
Respiratory distress syndrome	yes	yes	yes	yes
Mechanical ventilation in days	8	6	1	13
High flow nasal cannula in days	54	47	19	46
Bronchopulmonary dysplasia	yes	yes	no	yes
Patent ductus arteriosus	no	no	no	yes, pharmacolo-gically closed
Retinopathy of prematurity	no	no	grade 2, zone II	no
Septicaemic episodes	staphylococcus capitis (late)	none	candida gilliermondii, *Candida parapsilosis*, coagulase-negative staphylococcus – 2 episodes (late)	e.coli, ureoplasma ureoliticum (early), coagulase-negative staphylococcus (late)
Cranial ultrasound^c^	Bilateral IVH grade III	Bilateral IVH grade III Left APHI	Bilateral IVH grade III	Bilateral IVH grade III Post-haemorrhagic hydrocephalus (resolved)

Key: IVH = intraventricular haemorrhage; APHI = apparent periventricular haemorrhagic infarction

^a^Biamniotic bichorionic twin brothers;

^b^twin brother died shortly after delivery;

^c^classification according to Volpe (2018)

### Recordings and Assessment of GMs

Video recordings commenced 3 weeks after birth and were repeated at least thrice by the time the infants were discharged from hospital. In addition, each infant was videotaped twice during the first month after term and once at 14 weeks postterm age (Table 2). The infants were recorded lying in supine during periods of activity (preterm age), or active wakefulness (postterm age), wearing minimal clothing for 3 to 5 min (Einspieler et al. 2004). GMs were classified as normal or abnormal according to the Gestalt perception of the age-specific GM pattern: GMs during preterm age, around term and during the first month postterm age were scored as (a) “normal” if the movement sequence, amplitude, speed and intensity were variable; (b) “poor repertoire” (PR) if the sequence of movement components was monotonous and the amplitude, speed and intensity lacked the normal variability; (c) “cramped-synchronised” (CS) if GMs lacked the usual smoothness and appeared rigid, with limbs and trunk muscles contracting almost simultaneously and then relaxing almost simultaneously; or (d) “chaotic” (Ch) if the amplitude was large, the speed high, and GMs consistently appeared to be abrupt (Einspieler et al. 2004, 2015; Einspieler and Prechtl 2005).

**Table 2:** Individual developmental trajectories of GMs

Post-menstrual age in weeks	27	28	29	30	31	32	33	34	35	36–37	38–39	40–41	42–45	54
Case 1		B			PR^C^	PR^C^		CS		**CS**	**CS**	**PR**^**C**^	**PR**	F+ AR
Case 2		B			PR^C^	Ch		CS		**CS**	**CS**	**CS**	**PR**^**C**^	F+ IR
Case 3	B			PR		PR^C^			CS	**CS**	**CS**	**CS**	**PR**^**C**^	F+ IR
Case 4		B			PR^C^		CS		**CS**		**CS**	**PR**^**C**^	**PR**	AF IR

Key: AF = abnormal fidgety movements; AR = age-appropriate motor repertoire (Einspieler et al. 2004, 2008); B = birth; Ch = chaotic pattern of GMs; CS = cramped-synchronised pattern of GMs; F + =presence of normal fidgety movements; IR = age-inappropriate motor repertoire (Einspieler et al. 2004, 2008); PR = poor repertoire of GMs; PR^C^ = poor repertoire of GMs with cramped components

The period in bold indicates intensive intervention by the application of the MIT-PB procedure

At 14 weeks postterm, the age-specific form of GMs – fidgety movements – was scored as (a) “normal” if small movements of moderate speed with variable acceleration of the neck, trunk and limbs in all directions were present; (b) “abnormal” if the GMs looked like normal fidgety movements though with a greater amplitude, speed and jerkiness; or (c) “absent” if no fidgety movements were observed (Prechtl et al. 1997; Einspieler et al. 2004; Einspieler and Prechtl 2005).

GMs were scored by two certified scorers (M.S. and C.E) on the basis of standard GMA video recordings. At 14 weeks postterm age the concurrent movement repertoire, which includes wiggling-oscillating and swiping arm movements, kicking, movements towards the midline (hand-to-mouth contact, hand-to-hand contact, foot-to-foot contact), and antigravity movements (legs lift with or without hand-to-knee contact) (Einspieler et al. 2008), was scored as “age-appropriate” – or “age-inappropriate”, if movements towards the midline and antigravity movements were lacking. Interrater reliability was Kappa = 1.0 in the GMA and the assessment of the concurrent movements. C.E. was blinded to the clinical history of the cases.

### MIT-PB Procedure: Attempting to Optimise the Pattern of GMs

GMs are produced by a central pattern generator (CPG), a neural network which is probably located in the brain stem. In order to lend variability to the motor output, supraspinal projections and the sensory feedback modulate the CPG activity (Einspieler et al. 2004, 2016). In case of brain injury, the CPG modulation by supraspinal projection is limited. Hence, we assume that one way of modulating CPG activity is to move the infant’s limbs in a variable mode so as to enhance the variability of sensory feedback.

A few days after CS GMs were diagnosed, the MIT-PB [Movement Imitation Therapy for Preterm Babies] procedure was started (the duration is highlighted in bold in Table 2). MIT-PB was launched by the first author, a neonatologist, in cooperation with a developmental physiotherapist (both are henceforth referred to as “therapists”). The procedure was the following: the instant an infant showed CS movements, the therapists (or a therapist and a parent) intervened by gently guiding the infants’ limbs so as to manoeuvre and smoothen their movements, thereby imitating normal GM sequences as closely as possible. Simultaneous extension followed by simultaneous relaxation of the limbs is transformed to smooth and fluent movements by the therapists, i.e. superimposing a variable sequence of movements mimicking normal GMs; e.g., increasing rotatory components, waxing and waning, as well as changing the amplitude of the movements to enhance variability of the repertoire. The therapists had to avoid stressful movements at all times in order to keep the infants calm. Each intervention lasted for at least 10 min. As seen in normal GMs, mimicry GMs were paused in between and when the infants next cramped synchronised movement started, resumed. If an infant started to cry the intervention was paused; the infant was calmed by increased body contact and cuddling. The intervention was repeated at least 5 times per day and the parents were instructed and trained from the very beginning to keep intervening after discharge from the hospital. In this way MIT-PB was maintained until 5 weeks postterm age, i.e. 11 weeks for Cases 1 and 2, 10 weeks for Case 3, and 12 weeks for Case 4 (see Table 2). After discharge from the hospital (at 38 or 39 weeks postmenstrual age) the parents continued to apply MIT-PB as described above; the infants were seen by the therapists on a weekly basis in order to supervise and adjust the parents’ manoeuvres.

In addition, parents were trained to handle and posture their infant, which is a standard procedure for all preterm infants born at Carmel Hospital. From 2 to 9 months, all four infants received intervention once a week in accordance with the Neurodevelopmental Treatment (Bobath) concept; Case 2 received weekly treatment until 12 months. Subsequently, each child was seen at least once per month by a neurodevelopmental therapist.

### Neurological Examination and Follow-up

During the first 2 years of life, serial clinical assessments included a neurological examination based on Amiel-Tison and Grenier (1983). At the end of the second year, the Bayley Scales of Infant Development, second edition (BSID-II; Bayley 1993) was applied as the third edition was not yet standardized for Israel. In the 5th year of life, the Miller Assessment for Preschoolers (Miller 1988) was used for developmental evaluation (see Table 3).

**Table 3:** Neurodevelopmental assessment

	Case 1	Case 2	Case 3	Case 4
Serial neurological examinations from 3 months postterm age onwards^a^	Normal	Hypotonia in the trunk until 12 months of age, thereafter improved. At 4 years and 9 months: mild asymmetry of tendon reflexes (R > L)	Hypotonia in the trunk until 10 months of age, thereafter normal	Normal
Bayley Scales of Infant Development (second edition) ^b^	MDI = 115 (CI 107–121) PDI = 108 (CI 99–115) at 23 months	MDI = 119 (CI =111–125) PDI = 89 (CI 79–99) at 23 months	MDI = 122 (CI 112–128) PDI = 124 (CI 111–131) at 23 months	MDI = 122 (CI 110–130) PDI = 125 (CI 110–130) at 20 months
Miller Assessment for Preschoolers^c^	Total score = 74 Performance indices: FI = 63 CoI = 53 VI = 48 N-VI = 53 CTI = 50	Total score = 74 Performance indices: FI = 63 CoI = 53 VI = 48 N-VI = 53 CTI = 50	Total score = 64 Performance indices: FI = 42 CoI = 53 VI = 48 N-VI = 53 CTI = 50	Total score = 74 Performance indices: FI = 63 CoI = 53 VI = 48 N-VI = 53 CTI = 50
	at 4 years 9 month	at 4 years 9 month	at 4 years 4 month	at 4 years 1 month

Key: MDI = mental developmental index; PDI = psychomotor developmental index; FI=Foundations Index (sense of position and movement, sense of touch, components of basic movements pattern); CI=Confidence Interval; CoI=Coordination Index (gross and fine motor abilities, oral motor abilities); VI=Verbal Index (speech and language abilities); N-VI = Non-Verbal Index (non-verbal cognitive abilities, attention, perception, memory); CTI=Complex Tasks Index (combination of sensory, motor, cognitive abilities)

^a^according to Amiel-Tison and Grenier (1983)

^b^in 2014, the Bayley-III was not yet standardized for Israel

^c^according to the revised Manual (1988): scores in the range 50 and above indicate average or above average ability

## Results

All infants showed PR GMs at their first video recording 3 weeks after birth. During these first recordings, cramped components, especially in the lower extremities, could already be observed in Cases 1, 2 and 4. Case 3 only demonstrated cramped components 5 weeks after birth, at 32 weeks postmenstrual age. In Case 2, PR GMs were followed by Ch GMs. At 33 to 35 weeks (i.e. 5 to 8 weeks postnatal), all infants demonstrated CS GMs. Two to 3 days later, MIT-PB was launched as described above. CS GMs continued to be present until term-equivalent age. Then GMs improved to PR, although cramped components could be observed every now and then (Table 2).

At 14 weeks postterm age, Cases 1, 2 and 3 showed normal fidgety movements, albeit only intermittently and poorly expressed. Case 4 had abnormal fidgety movements. The concurrent motor repertoire of Case 1 was age-appropriate. Cases 2, 3 and 4 did not show arm and leg movements towards the midline. In Case 2 the asymmetric tonic neck posture was still predominantly present (Table 2, right column).

At 24 months, all individuals were neurologically normal. Also all BSID-II developmental indices (both mental and psychomotor) were within the normal range; most of them even above the first standard deviation (Table 3). At 4 to 5 years, all cases had normal scores in the Miller Assessment for Preschoolers (Miller 1988).

## Discussion

Very preterm infants often have PR GMs during their first weeks of life (Einspieler et al. 2015; Olsen et al. 2015), but longitudinal GMA reveals that in most preterm infants the quality of GMs normalises by term age (Prechtl et al. 1997; Nakajima et al. 2006). If, however, GMs remain abnormal until 3 to 5 months postterm age, the infant has a high risk for an adverse neurodevelopmental outcome (e.g., Prechtl et al. 1997; Ferrari et al. 2002; Nakajima et al. 2006; Beccaria et al. 2012; Spittle et al. 2013; Einspieler et al. 2016). Especially CS GMs, if consistently present and concurring with severe brain injury, are predictive for the development of spastic cerebral palsy (e.g., Prechtl et al. 1997; Ferrari et al. 1990, 2002; Bruggink et al. 2009; Yang et al. 2012; Einspieler et al. 2013; Novak et al. 2017). As far as we can tell, the neurological outcome may only be normal if the CS GMs are transient and the brain injury is less severe (Ferrari et al. 2002).

The four cases of our intervention approach had consistent CS GMs (lasting 5 to 7 weeks, Table 2) but already performed significantly better within the first month post term. All participants developed fidgety movements, although the quality was abnormal in Case 4. To the best of our knowledge, such normalisation of GMs after a comparably long period of CS GMs is unprecedented. Case 1 even had an age-appropriate normal repertoire of concurrent movements, though Cases 2 to 4 showed no movements towards the midline at the 14-week recording. The lack of movements towards the midline is in accordance with a recently published study on extremely preterm infants who showed fewer antigravity movements at 12 weeks postterm age than their peers born at term (Fjørtoft et al. 2016).

For about 25 years, the Prechtl GMA has enabled us to reliably identify infants at high risk for later neurological dysfunctions at a very early stage. GMA is especially powerful if combined with MRI at term age (e.g., Spittle et al. 2009; Ferrari et al. 2011; Bosanquet et al. 2013). On the other hand, there is still few evidence to suggest that such early identification can entail effective early intervention.

Neither a single-session of physiotherapy (Kepenek-Varol et al. 2019) nor a parent-administered early intervention programme applied between 34 and 36 weeks’ gestational age (Fjørtoft et al. 2017) had an effect on fidgety movements and the concurrent motor repertoire. Craniosacral therapy applied in the neonatal intensive care unit did also not change the GM quality (Raith et al. 2016). By contrast, an early intervention programme launched already at the 3rd day of life in preterm infants improved the quality of fidgety movements but had no immediate effect on the GMs during preterm and at term age (Ma et al. 2015). Recently, a family-based intervention applied in preterm infants with abnormal GMs revealed positive effects on motor function (Kara et al. 2019). Together with our own promising results, this adds to recent developments promoting family centred intervention programmes such as GAME (Morgan et al. 2016b), COPCA (Hadders-Algra et al. 2017), LEAP-CP (Benfer et al. 2018), or EI-SMART (Hutchon et al. 2019).

We are aware that our pilot study comes along with certain limitations. Our sample is too small to draw general conclusions about the effect of MIT-PB. At this point, it should be seen as starting point and method development that needs to be replicated in larger samples. In addition, intervention studies, as the one reported here, may bear co-intervention effects. MIT-PB was done complementary to neurodevelopmental therapy (NDT) as treatment as usual (TAU) procedure. Future large-scale studies are needed to evaluate the effect of MIT-PB in its own right.

Our report is one of the first with a focus on intervention focusing on movement optimization during preterm age and its effect on CS GMs. It is remarkable that three of four children developed normal fidgety movements after a long period of CS GMs and that all four children had a normal neurodevelopmental outcome. The successful application of our MIT-PB procedure might encourage researchers and clinicians to continue testing the impact of mimicking normal and variable GM sequences on the neurodevelopmental outcome, not to mention the benefits of such a simple, parent-mediated intervention method.
